# A novel human truncated IL12rβ1-Fc fusion protein ameliorates experimental autoimmune encephalomyelitis via specific binding of p40 to inhibit Th1 and Th17 cell differentiation

**DOI:** 10.18632/oncotarget.5164

**Published:** 2015-09-04

**Authors:** Wei Guo, Chen Wang, Xin Wang, Cheng Luo, Dongmei Yu, Yuheng Wang, Yucong Chen, Wen Lei, Xiangdong Gao, Wenbing Yao

**Affiliations:** ^1^ State Key Laboratory of Natural Medicines, School of Life Science and Technology, China Pharmaceutical University, Nanjing, 210009 China

**Keywords:** Immunology and Microbiology Section, Immune response, Immunity, cytokine-blocking, binding specificity, molecular mechanism, central nervous system, autoimmune disease

## Abstract

Interleukin (IL)-12 and IL-23 respectively driving polarization of T helper (Th) 1 and Th17 cells has been strongly implicated in the pathogenesis of both multiple sclerosis (MS) and experimental autoimmune encephalomyelitis (EAE). In this study, we first constructed, expressed and purified a novel human truncated IL12rβ1-Fc fusion protein (tIL12rβ1/Fc) binding multiple forms of the p40 subunit of human IL-12 and IL-23. tIL12rβ1/Fc was found to effectively ameliorate MOG_35–55_-induced EAE through reducing the production of Th1- and Th17-polarized pro-inflammatory cytokines and suppressing inflammation and demyelination in the focused parts. Moreover, tIL12rβ1/Fc suppressed Th1 (IFN-γ^+^ alone) and IFN-γ^+^ IL-17^+^ as well as the population of classic Th17 (IL-17^+^ alone) cells *in vivo*. Furthermore, tIL12rβ1/Fc ameliorated EAE at the peak of disease *via* the inhibition of STAT pathway, thereby causing a prominent reduction of RORγt (Th17) and T-bet (Th1) expression. Notably, tIL12rβ1/Fc could increase the relative number of CD4^+^ Foxp3^+^ regulatory T cells. These findings indicates that tIL12rβ1/Fc is a novel fusion protein for specific binding multiple forms of p40 subunit to exert potent anti-inflammatory effects and provides a valuable approach for the treatment of MS and other autoimmune diseases.

## INTRODUCTION

Multiple sclerosis (MS) is a complex neurological disorder of the central nervous system (CNS) characterized by a variety of clinical symptoms that result in a range of progressive impairments and disability. To date, some treatments have been developed on slowing disease progression and alleviating symptoms but not a cure for MS [1]. The underlying cause of MS has only partially been elucidated, but ample findings indicate a central role for T helper (Th) cells in the pathogenesis of this disease. Experimental autoimmune encephalomyelitis (EAE), a T cell-correlated demyelinating disorder of CNS in mice, has been served as an animal model to assess the fate of MS progression for years [2]. Historically, both MS and EAE were considered as Th1-correlated diseases, dominated by the IL-12-polarized, interferon-γ (IFN-γ)-producing effector CD4^+^ T cells [3, 4]. Recent studies suggested that Th17, a distinct CD4^+^ T cell subset characterized by the secretion of IL-17 and the dependence on IL-23 for its expansion, exhibits greater pathogenicity for autoimmune inflammation of the brain [5, 6]. Importantly, that both Th1 and Th17 cells have been confirmed to induce inflammatory disease and distinct types of EAE [7, 8].

As is well known, naive T cells cultured *in vitro* under polarized conditions generally develop into specific groups, including those that produce IFN-γ (“Th1”), and those that produce IL-17 (“Th17”), upon activation [9–11]. Recently Th17/Th1 cells producing both IL-17 and IFN-γ from inflamed tissues and human peripheral blood were named [12]. The Th17/Th1 cells not only express RORγt, but also the master Th1-correlated transcription factor, T-bet. Moreover, the stimulation of human Th17 clones in the presence of IL-12 decrease RORγt and increase the expression of T-bet, enabling these Th17 cells to produce IFN-γ [13]. IL-23 might drive the expression of IFN-γ in Th17 cells without a direct correlation with T-bet [14]. Furthermore, the IFN-γ/IL-17A double-positive cells were enriched in the target organs of several autoimmune disease models including EAE [15]. Interestingly, IL-12 induces Th1 cells, while IL-23 promotes the generation of Th17 cells. IL-12 and IL-23 share the common p40 subunit. It was reported that blocking IL-12/23-p40 inhibited the receptor-binding of both IL-12 (a heterodimer of p35 and p40) and IL-23 (a heterodimer of p19 and p40). Notably, ustekinumab, a humanized monoclonal antibody inhibiting p40 showed marked clinical efficacy for the treatment of chronic inflammatory disorders such as psoriasis and psoriatic arthritis [16, 17]. However, ustekinumab was ineffective against clinical MS. Therefore, it is necessary to develop a new approach to inhibit IL-12 and IL-23 and prevent polarization of Th1 and Th17 cells for the amelioration of both MS and EAE.

In the present study, we utilized the extracellular soluble region of the p40 receptor to design a novel human truncated IL12rβ1-Fc fusion protein (tIL12rβ1/Fc). We found that tIL12rβ1/Fc specifically and effectively bound the p40 subunit of IL-12/23. tIL12rβ1/Fc indeed ameliorated MOG_35–55_-induced EAE through reducing the production of Th1- and Th17-polarized pro-inflammatory cytokines and suppressing inflammation and demyelination in the focused parts. Furthermore, tIL12rβ1/Fc reduced transcript factor RORγt (Th17) and T-bet (Th1) expressions. Moreover, tIL12rβ1/Fc could increase the relative number of CD4^+^ Foxp3^+^ regulatory T cells. These findings indicate that tIL12rβ1/Fc could be a novel fusion protein to exert anti-inflammatory effects and ameliorate MS and other autoimmune diseases.

## RESULTS

### Construction, expression, and purification of tIL12rβ1/Fc

**Figure 1:**
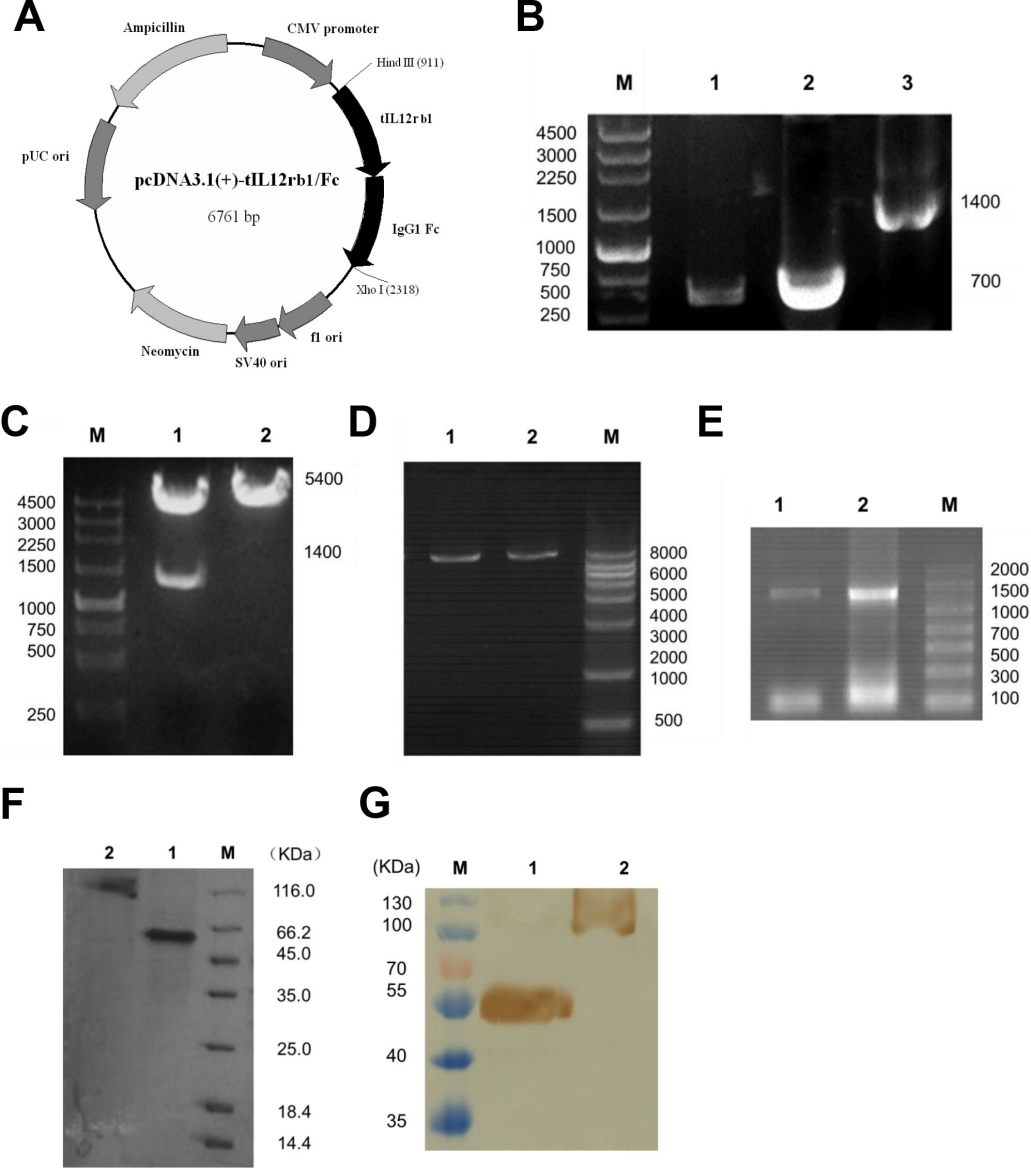
Construction, expression, and purification of tIL12rβ1/Fc **A.** Plasmid map of the eukaryotic expression plasmid containing human truncated IL-12rβ1 receptor (pcDNA3.1(+)-tIL12rβ1/Fc). The gene sequence encoding tIL12rβ1/Fc was inserted into pcDNA3.1(+) vector at the corresponding restriction sites *Hind* III and *Xho* I. **B.** PCR products of tIL12rβ1/Fc recombinant gene. Lane 1, tIL12rβ1/Fc gene sequence containing the signal peptide. Lane 2, IgG1 Fc fragment. Lane 3, recombinant tIL12rβ1/Fc fused gene product using overlap PCR. M, DNA molecular weight markers, bp. **C.** Correctly constructed plasmid was verified by digestion at the *Hind* III and *Xho* I sites. Lane 1, pcDNA3.1(+)-tIL12rβ1/Fc after digestion. Lane 2, pcDNA3.1(+) -tIL12rβ1/Fc before digestion. M, DNA molecular weight markers, bp. **D.** Plasmids were linearized for cell transfection using *Pvu* I. Lane 1 and 2, linearized pcDNA3.1(+)-tIL12rβ1/Fc. M, DNA molecular weight markers, bp. **E.** Total RNA of the two strains were identified by RT-PCR with primers F1 and F4. **F.** SDS-PAGE analysis of the purified tIL12rβ1/Fc fusion protein using Protein A column. Lane 1, purified tIL12rβ1/Fc fusion protein at reduced state. Lane 2, purified tIL12rβ1/Fc fusion protein at non-reduced state. M, protein molecular weight markers, KDa. **G.** Western blot analysis of tIL12rβ1/Fc using mAbs against human IL12rβ1. Lane 1, purified tIL12rβ1/Fc fusion protein at reduced state. Lane 2, purified tIL12rβ1/Fc fusion protein at non-reduced state. M, protein molecular weight markers, KDa.

We first chose the extracellular soluble region of IL-12/ 23 receptor fusing with Fc fragment for stronger stability and easy preparation to bind the p40 subunit of IL-12/IL-23. To construct the eukaryotic expression plasmid, the tIL12rβ1 and IgG1 Fc genes amplified using RT-PCR were fused and then cloned into pcDNA3.1(+) at the restriction digestion sites *Xho* I and *Hind* III (Fig. 1A and 1B). The correct sequence of tIL12rβ1/Fc fusion gene was confirmed by restriction digestion (Fig. 1C) and DNA sequencing. Next, the correctly constructed plasmid was linearized (Fig. 1D) and then transfected into CHO-K1 cells using electric transfection method. A stable expression cell line was obtained after the screening with the addition of 400 μg/mL G418 sulfate and finally verified using ELISA and RT-PCR (Fig. 1E). Later, the medium was changed to SFM medium without FBS and the cell cultures containing tIL12rβ1/Fc protein were purified by Protein A chromatography. The eluted protein was obtained with a purity of approximately 90% examined by SDS-PAGE (Fig. 1F) and recognized by anti-hIL12rβ1 mAbs using Western blot (Fig. 1G). The data indicated that tIL12rβ1/Fc mainly exists in a monomeric form at the reduced state while forms a dimer at the non-reduced condition (Fig. 1F and 1G). Of note, the reduced protein was visibly found near 55 kDa band, not accordance with its theoretical molecular weight of 50 kDa, presumably due to the glycosylation modifications in the eukaryotic expression system (Fig. 1F and 1G).

**Figure 2:**
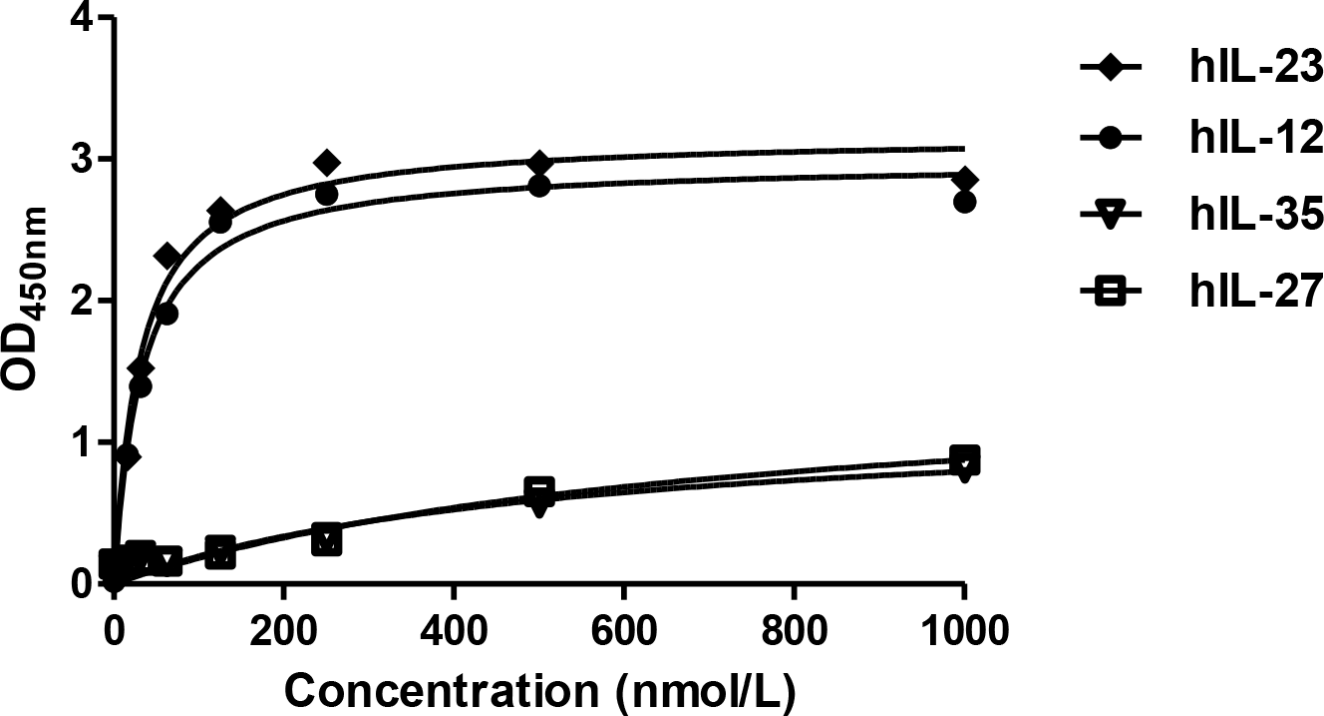
Binding affinity of tIL12rβ1/Fc protein to hIL-12/IL-23 and hIL -27/IL-35 Binding affinity of tIL12rβ1/Fc protein to human IL-12 and human IL-23 were examined by direct binding ELISA. Meanwhile, the binding assays of tIL12rβ1/Fc protein to human IL-27 and IL-35 were also conducted as controls to confirm the binding specificity, showing no cross-reactions with our molecule.

The binding affinities of tIL12rβ1/Fc protein to human IL-12 and IL-23 were determined by direct binding ELISA to give EC_50_ values of 30.20 and 32.74 nmol/L, respectively (Fig. 2). Meanwhile, the binding assays of tIL12rβ1/Fc protein to human IL-27 and IL-35 were also conducted as controls to confirm the binding specificity, showing no cross-reactions with our molecule (Fig. 2). To further assess the binding specificity, much weaker binding affinity of tIL12rβ1/Fc with mouse IL-12 and IL-23 was observed due possibly to the sequence difference (Fig. S1). Together, the binding assays unambiguously confirmed the specific binding between tIL12rβ1/Fc and human IL-12 or IL-23, indicating that the eukaryotically expressed tIL12rβ1/Fc exhibits desired biological activity.

### tIL12rβ1/Fc ameliorated symptoms of MOG_35–55_-induced in correlation with decreased production of Th1 and Th17 cytokines

**Figure 3:**
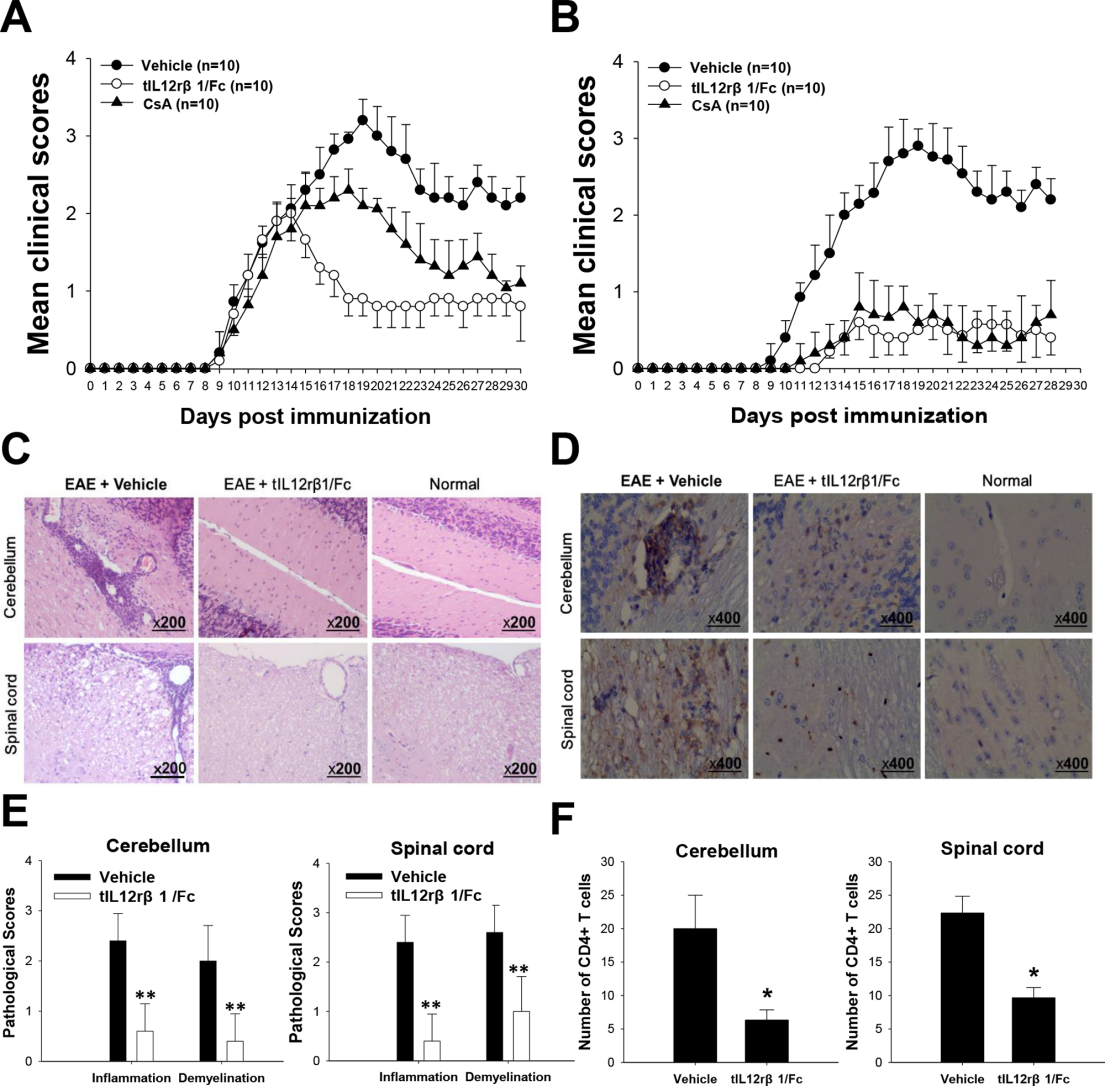
Amelioration of EAE by tIL12rβ1/Fc treatment The tIL12rβ1/Fc or vehicle (saline) was administered via tail vein injection at 2.5 mg/kg and CsA was administered i.g. as positive control at 1.5 mg/kg. The regimen started 9 days after immunization as therapeutic regimen **(A)** or 3 days before immunization as preventive regimen **(B). C.** H&E staining analysis of brains and spinal cords obtained from normal mice or mice treated with vehicle or tIL12rβ1/Fc at day 30 post-immunization (therapeutic protocol). **D.** Immunohistochemistry for CD4^+^ T cell infiltration of brains and spinal cords obtained from normal mice or mice treated with vehicle or tIL12rβ1/Fc at day 30 post-immunization (therapeutic protocol). **E.** Pathology scores of inflammation and demyelination in brains and spinal cords. **F.** The number of CD4^+^ T cells in spinal cords and brains. Data are expressed as mean ± SD (*n* = 9). *p < 0.05; **p < 0.01.

The treatment effects of tIL12rβ1/Fc were first investigated in EAE mouse model, showing that the administration of tIL12rβ1/Fc can remarkably ameliorate EAE with several lines of evidence. In the treatment regimen, tIL12rβ1/Fc administration started from disease onset in animals (day 9 post-immunization) led to a significant decrease in disease severity measured by the mean clinical score or mean maximum clinical score compared to vehicle control (Fig. 3A and Table 1). In the prophylactic treatment regimen, tIL12rβ1/Fc administration started from day 3 before immunization was more effective in decreasing the clinical score of EAE mice (Fig. 3B and Table 1). Furthermore, the prophylactic dosing of tIL12rβ1/Fc also resulted in delayed disease onset (Fig. 3B).

**Table 1:** Clinical features of EAE in mice in the administration of vehicle, tIL12rβ1/Fc or CsA

Group	Treatment regimen		Preventive regimen	
	Incidence, %	Mean maximal score	Incidence, %	Mean maximal score
Vehicle	100	3.25 ± 0.35	100	2.95 ± 0.44
tIL12rβ1/Fc	100	1.90 ± 0.46^***^	70	1.10 ± 0.62^***^
CsA	100	2.30 ± 0.43^***^	80	1.20 ± 0.75^***^

Values are expressed as mean ± SD

*** *p* < 0.001 compared with vehicle control.

Cyclosporin A (CsA), an immunosuppressive agent commonly used in the clinical therapy for autoimmune diseases, was used in the treatment of EAE as a positive control. In our experiments, the tIL12rβ1/Fc treatment showed similar effectiveness as CsA under the preventive regimen (Fig. 3B and Table 1) and even more effective than CsA under therapeutic protocol (Fig. 3A and Table 1). In addition, CsA treatment caused obvious loss of body weight of EAE mice. By contrast, tIL12rβ1/Fc-treated mice had no obvious effect in body weight over the course of the treatment. This suggested that tIL12rβ1/Fc may possess less safety concerns. (Fig. S2). The tIL12rβ1/Fc administration markedly attenuated demyelination and CD4+T cell infiltration in stimulated spinal cords and brain sections compared to that of the vehicle group of EAE mice (Fig. 3C and 3D). The pathological scores of cerebellum and spinal cord in vehicle-treated and tIL12rβ1/Fc-treated mice differed significantly (Fig. 3E). In addition, the infiltration of CD4^+^ T cells was substantially decreased in response to tIL12rβ1/Fc treatment (Fig. 3F).

**Figure 4:**
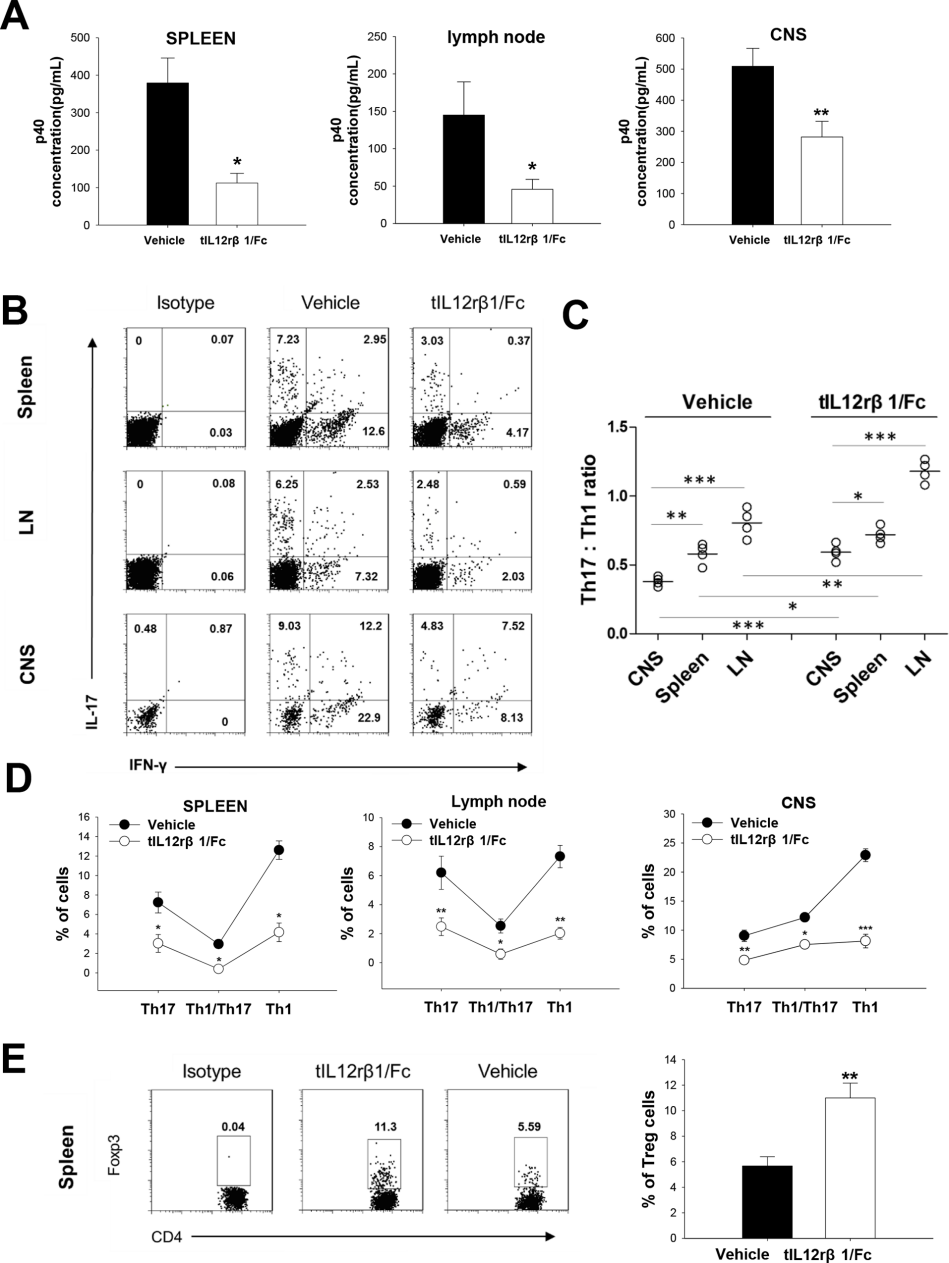
Downregulation of Th1 and Th17 cells by tIL12rβ1/Fc treatment Splenocytes, lymph nodes cells and CNS MNCs of tIL12rβ1/Fc- or vehicle-treated EAE mice at day 18 post-immunization (therapeutic protocol) were isolated. **A.** Supernatants derived from splenocytes, lymph nodes cells and CNS MNCs reactivated with MOG_35–55_ (20 μg/mL) for 24 h were analyzed for the level of p40 (mean ± SD; *n* = 5). **B.** The percentage of Th1, Th1/Th17 and Th17 cells in the CD4^+^ lymphocyte gate were analyzed by intracellular staining of IFN-γ and IL-17, following stimulation with MOG_35–55_ (20 μg/mL) for 24 h. **C.** The Th17:Th1 ratios in CNS, LNs and Spleens were observed for each group. Correlation of Th17:Th1 ratios with vehicle treatment and that with tIL12rβ1/Fc treatment was significant (*p* < 0.05) (*n* = 4). **D.** Percentages of cells positive expression with these antigens in spleen, LN or CNS are expressed as mean ± SD (*n* = 4). **E**. The frequency of regulatory T cells in the CD4^+^ lymphocyte gate were analyzed by intracellular staining of Foxp3 following stimulation with MOG_35–55_ (20 μg/mL) for 24 h. Percentage of Foxp3^+^ cells in spleen is expressed as mean ± SD (*n* = 4). *p < 0.05; **p < 0.01; ***p < 0.001.

As expected, the therapeutic effects of tIL12rβ1/Fc were associated with a significant suppression of p40 concentration in mice (Fig. 4A). This suppression led to a prominent reduction in the percentage of Th1, Th17 cells as well as the pathogenic Th17/Th1 cells in spleens, LNs and brains compared to the vehicle control (Fig. 4B and 4D). In addition, the Th17/Th1 ratio was significantly decreased in the lesion CNS compared with that in the spleens and LNs in EAE mice(Fig. 4C). An obvious elevation of the Th17/Th1 ratio after tIL12rβ1/Fc administration was also observed (Fig. 4C). tIL12rβ1/Fc treatment also caused an improvement on the relative number of CD4^+^ Foxp3^+^ regulatory T cells (Fig. 4E).

**Figure 5:**
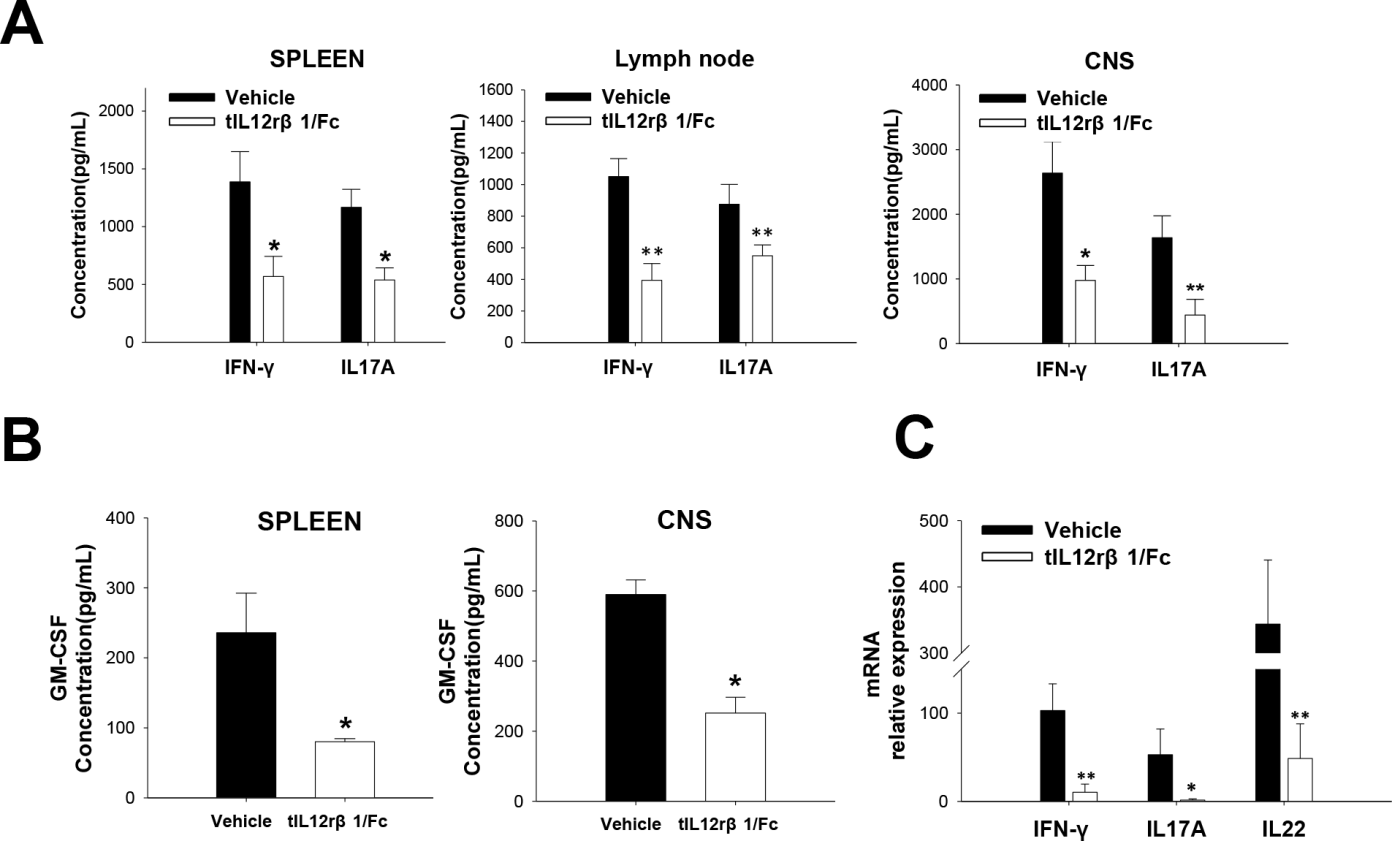
Suppression of related cytokines by tIL12rβ1/Fc treatment Splenocytes, lymph nodes cells and CNS MNCs of tIL12rβ1/Fc- or vehicle-treated EAE mice at day 18 post-immunization (therapeutic protocol) were isolated. **A.** Supernatants derived from splenocytes, lymph nodes cells and CNS MNCs reactivated with MOG_35–55_ (20 μg/mL) for 24 h were analyzed for the level of IFN-γ and IL-17A (mean ± SD; *n* = 5). **B.** Supernatants derived from splenocytes and CNS MNCs reactivated with MOG_35–55_ (20 μg/mL) for 24 h were also measured for the expression of GM-CSF. **C.** mRNA level of IFN-γ, IL-17A and IL-22 from the same CD4+ splenocyte preparations were analyzed by quantitative real-time PCR. Data are representative of three independent experiments. *p < 0.05; **p < 0.01.

At the same time, the production of both Th1-secreated cytokine IFN-γ and Th17-secreated cytokine IL-17A were remarkably reduced by tIL12rβ1/Fc treatment (Fig. 5A). Given the critical roles of cytokines in MS and EAE pathogenesis, the expression of granulocyte macrophage-colony stimulating factor (GM-CSF) was also examined, and GM-CSF level was greatly reduced after tIL12rβ1/Fc treatment (Fig. 5B). In addition, the mRNA levels of Th1- and Th17-correlated factors including IFN-γ, IL-17A and IL-22 were also significantly decreased compared to that of control group of EAE mice (Fig. 5C).

### tIL12rβ1/Fc treatment changed Th1 and Th17 cell differentiation *in vitro*

**Figure 6:**
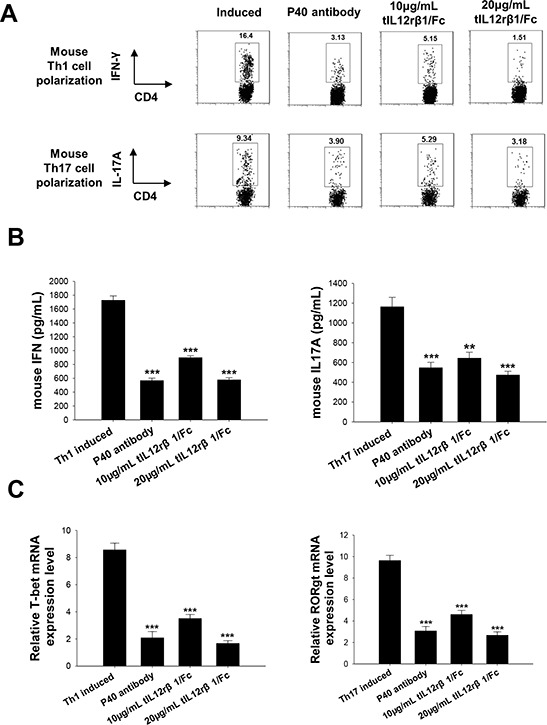
Role of tIL12rβ1/Fc in mouse Th1 and Th17 cell differentiation Mouse naive CD4^+^ cells were differentiated into Th1 or Th17 cells in the presence of different concentrations of tIL12rβ1/Fc *in vitro*. **A.** The percentage of Th1 and Th17 cells in the CD4 subset were analyzed by intracellular staining of IFN-γ and IL-17A, respectively. **B.** Supernatants derived from cultured spleen cells, as above, were analyzed for the indicated cytokines. **C.** mRNA abundance of transcription factor RORγt and T-bet were also measured by quantitative real-time RT-PCR. Data are representative of three independent experiments. *p < 0.05; **p < 0.01; ***p < 0.001.

**Figure 7:**
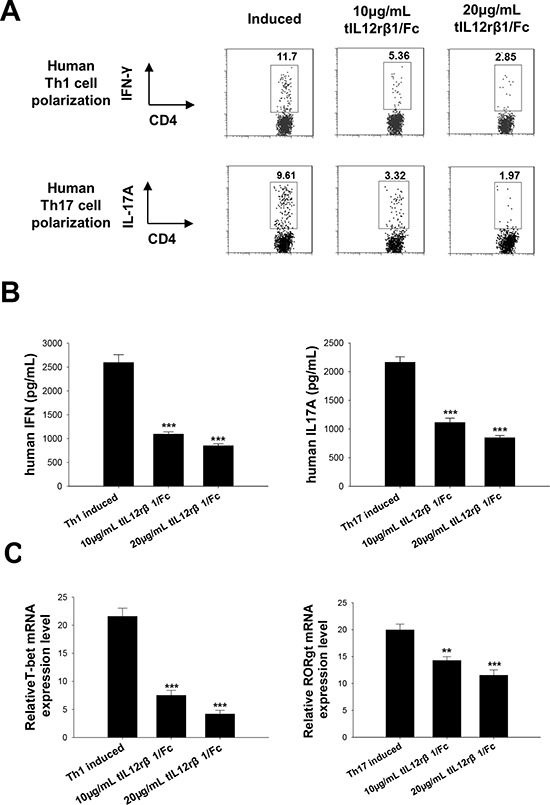
Role of tIL12rβ1/Fc in human Th1 and Th17 cell differentiation Human PBMCs purified by density gradient centrifugation using Histopaque were differentiated into Th1 or Th17 cells in the presence of indicated tIL12rβ1/Fc. **A.** Human Th1 and Th17 development in the CD4 gate in PBMCs were examined by Flow cytometry analysis. **B.** The level of secreted IFN-γ and IL-17A in cell culture supernatants were analyzed using ELISA method. **C.** mRNA expression of T-bet and RORγt from the same cell preparations were analyzed by quantitative real-time PCR. Data are representative of three independent experiments. **p < 0.01; ***p < 0.001.

To further delineate whether the effects from tIL12rβ1/Fc treatment were associated with Th1 or Th17 cell differentiation, purified naive mouse CD4^+^ T cells were cultured in the presence or absence of the recombinant protein *in vitro* under Th1- or Th17-polarizing conditions. Consistent with the effectiveness of tIL12rβ1/Fc in animal experiments, tIL12rβ1/Fc treatments had similar efficacy as the anti-mouse p40 antibody on inhibiting both mouse Th1 and Th17 cell differentiation (Fig. 6A). With the treatment of tIL12rβ1/Fc, cytokine levels of IFN-γ and IL-17A and mRNA expression of T-bet and RORγt were also remarkably decreased as shown in Fig. 6B and 6C. Furthermore, tIL12rβ1/Fc was also confirmed to suppress the development of human Th1 and Th17 cells *in vitro* (Fig. 7A) with a significant reduction of the cytokine secretion (Fig. 7B). By further examining the expression of Th1- and Th17- specific transcription factors, we observed tIL12rβ1/Fc could obviously decrease T-bet and RORγt mRNA in human PBMCs (Fig. 7C). The data collectively demonstrated that addition of tIL12rβ1/Fc inhibits both mouse and human Th1 and Th17 differentiation and the influence of tIL12rβ1/Fc on Th1 and Th17 differentiation seemed to act in a dose dependent manner.

### tIL12rβ1/Fc treatment inhibited STAT signaling pathway in the development of Th1 and Th17 cells

**Figure 8:**
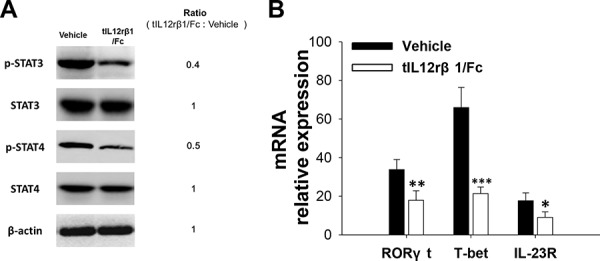
The inhibitory effect of tIL12rβ1/Fc on Th1 and Th17 in relation to STAT signaling pathway **A.** Purified CD4^+^ splenocytes from tIL12rβ1/Fc-treated mice or control EAE mice on day 18 post-immunization (therapeutic protocol) were analyzed by Western blot assay for the expression or phosphorylation of indicated proteins in the STAT pathway. **B.** The same CD4^+^ T cell preparations were measured for the mRNA abundance of RORγt, T-bet and IL-23R by quantitative real-time RT-PCR. Data are representative of three independent experiments. *p < 0.05; **p < 0.01; ***p < 0.001.

Due to the crucial roles of STAT family in the differentiation of Th cells, we first hypothesized that the receptor protein tIL12rβ1/Fc conducts regulatory effects on Th1 and Th17 development through direct inhibition on the STAT signaling pathway. To test it, splenic CD4^+^ T cells from tIL12rβ1/Fc-treated mice or control EAE mice at day 18 post-immunization (therapeutic protocol) were analyzed by Western blotting for the expression or phosphorylation of indicated proteins in the STAT signaling pathway. As illustrated in Fig. 8A, the expression of phosphorylated p-STAT4 from Th1 cells and p-STAT3 from Th17 cells was obviously decreased. In addition, the expression of two key transcription factors, T-bet for Th1 and RORγt for Th17 cells, was also significantly reduced (Fig. 8B). Additionally, the relative mRNA expression of IL23R in the tIL12rβ1/Fc treatment group was also decreased (Fig. 8B), which is in agreement to the reduction of STAT3 phosphorylation and RORγt expression.

### tIL12rβ1/Fc treatment suppressed NF-κB signaling pathway

**Figure 9:**
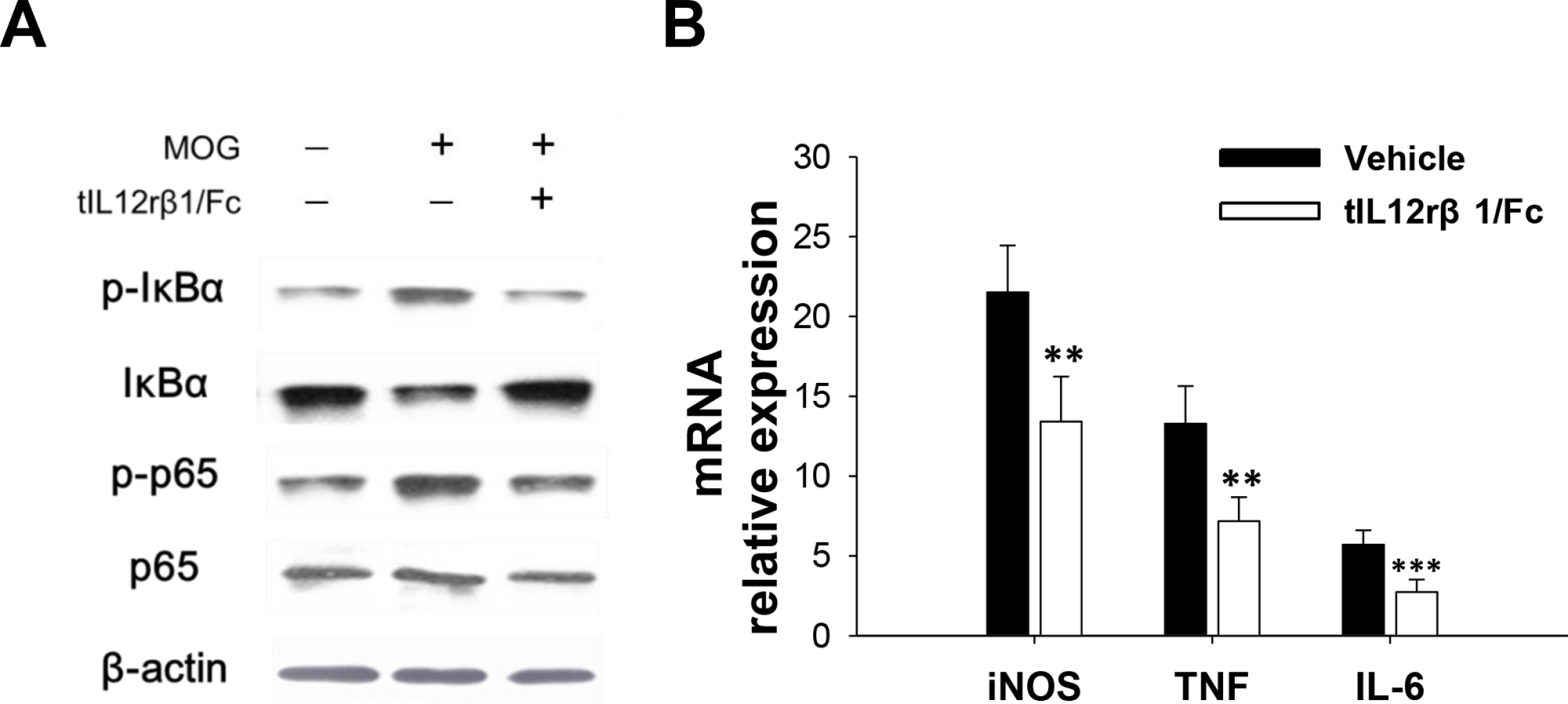
Inhibition of NF-κB signaling pathway by tIL12rβ1/Fc treatment **A.** Splenocytes from tIL12rβ1/Fc or vehicle-treated EAE mice on day 18 post-immunization (therapeutic protocol) were analyzed for phosphorylated IκBα, phosphorylated p65, IκBα and p65 by Western blot assay. **B.** Expression levels of NF-κB-regulated gene products including IL-6, TNF-α and iNOS were measured by quantitative real-time RT-PCR. Data are representative of three independent experiments. **p < 0.01; ***p < 0.001.

Since NF-κB signaling pathway is well-known to involve in the inflammatory process of EAE [18], we examined whether tIL12rβ1/Fc interferes with this vital pathway. After analyzing the levels of phosphorylated IκBα and p65 in splenocytes isolated from vehicle- or tIL12rβ1/Fc-treated EAE mice by Western blotting, it was revealed that expression of phosphorylated IκBα and phosphorylated p65 was decreased and expression of IκBα in tIL12rβ1/Fc-treated mice was increased compared to vehicle-treated mice (Fig. 9A). Consistent with the modified NF-κB activity, expression levels of NF-κB-regulated gene products including IL-6, TNF-α and iNOS were significantly reduced in splenocytes derived from the tIL12rβ1/Fc-treated mice compared to those from the vehicle group (Fig. 9B). All data strongly suggested the involvement of NF-κB signaling pathway in the development of EAE and the treatment effects by tIL12rβ1/Fc.

## DISCUSSION

During the past decade, highly potent biological agents targeting TNF-α [19], IL-6 [20] and p40 [21] to enhance or replace conventional immunosuppressive therapies have made significant advances in the treatment of autoimmune diseases. For example, ustekinumab, a fully human monoclonal antibody that binds the p40 subunit of IL-12 and IL-23, showed excellent efficacy in the treatment of psoriasis [21, 22]. However, in phase II clinical trials for MS, ustekinumab did not show obvious clinical improvements in MS patients [23, 24]. This could be due to the heterogeneous nature of the p40 subunit to prevent the antibody fully achieving its recognition and neutralizing effects. With regard to the ternary structure of p40, the p40 subunit of IL-12 can function in the form of both monomer and homodimer in addition to the formation of heterodimers with p35 and p19, and such a structural diversity has been proven to be critical for physiological function and disease progression [25]. The homodimeric form of IL-12 p40 subunit (p40_2_ or p80) is considered as a potentially strong candidate to trigger CNS inflammation and glial activation *via* the induction of iNOS [26]. Both monomer and homodimer of IL-12 p40 subunit have also been reported to contribute significantly to CNS inflammation *via* up-regulation of TNF-α in microglial cells [27], suggesting that different forms of p40 may independently play a key role in CNS inflammation and demyelination in EAE and MS. In addition, functional blocking monoclonal antibodies against p40 homodimer of IL-12 has been found to ameliorate clinical symptoms and disease progression of adoptive transfer EAE [28]. Nevertheless, given that recombinant IL-12 p80 can also bind to IL-12Rβ1 [29], the common receptor subunit shared by the p40 cytokine family simultaneously targeting IL-12, IL-23, both monomer and homodimer of p40 might be more advantageous than antibodies against p40 subunit alone and provide more effective treatments for MS and EAE. Moreover, utilizing extracellular binding domains of the receptors could be a more versatile and effective approach to bind different forms of the ligand, and consequently producing stronger biological responses. In the present work, the truncated human IL12rβ1-Fc fusion protein (tIL12rβ1/Fc) as a novel molecule with proved function was optimized through a series of screenings both in silico and in practice among a panel of related molecules to the p40 subunit.

In the EAE model, tIL12rβ1/Fc treatment obviously ameliorated EAE severity, as effective as or even more effective than CsA. Comparisons between tIL12rβ1/Fc and CsA group on body weight suggested that the specificity of tIL12rβ1/Fc targeting multiple forms of p40 subunit may result in safer outcomes when used in MS patients. Attenuation of demyelination and inflammation by tIL12rβ1/Fc further supported its potential curative effects on MS and potentially other autoimmune diseases. When given immediately after EAE induction (in the prophylactic dosing regimen), tIL12rβ1/Fc significantly delayed disease onset, suggesting that tIL12rβ1/Fc could suppress the early phase of the immune responses. In humans, Th17/1 cells were isolated from inflamed tissue of IBD patients and MS lesions [30], suggesting that these double producers are particularly pathogenic in tissue inflammation and autoimmunity. Previously, it was reported that Th17/Th1 and Th1 cells, rather than Th17 cells, play a critical role in disease progression [13]. In our study, tIL12rβ1/Fc suppressed Th1 (IFN-γ^+^ alone) and IFN-γ^+^ IL-17^+^ cells as well as the population of classic Th17 (IL-17^+^ alone) cells compared to vehicle. These results differ from the earlier experimental data conducting in the cGVHD model, indicating that the IL-17 single-positive (IFN-γ^−^) cells were not altered with the anti-p40 mAb treatment [31]. The reason for this difference may be due to the different pathogenesis of the two disease models. Until recently, it has not been clear whether the IL-17^+^ IFN-γ^+^ double producers arise from Th1 or Th17 cells. Annunziato F. et al. [12, 13, 32] provided evidence for the plasticity of human Th17 cells to shift to a Th1 profile, whereas Th1 cells seem to be unable to shift to Th17. In contrast, Kurschus et al. found that a highly pure Th1-cell population can convert into IFN-γ/IL-17 double-producing T cells *in vivo* [33]. Therefore, clarification of the origin of the double producer cells is of great importance with regard to the pathogenetic process of autoimmune, as well as of other chronic inflammatory disorders. From our experimental data, we found that the Th17:Th1 ratio is much higher in the peripheral lymphoid organs such as spleens and LNs than CNS, suggesting that the conversion of large numbers of pure Th17 cells into IFN-γ^+^ IL-17^+^ cells in CNS is essential in EAE. Also, our observation on the elevation of Th17:Th1 ratio after tIL12rβ1/Fc treatment suggested a shift of the “alternative” Th17 cells to “classical” Th17 cells, which is supported by the reduction of mRNA level of IL-22.

IL-12 is crucial for Th1 cell differentiation through STAT4 pathway and the activation of an unique transcription factor named T-bet [34, 35]. STAT3 and RORγt are reported to be necessary for differentiation of the Th17 lineage and sufficient to direct the expression of the hallmark cytokines of this lineage [36, 37]. Similar to these results, tIL12rβ1/Fc in this study decreased STAT4 phosphorylation and T-bet expression during Th1 differentiation and reduced STAT3 phosphorylation and RORγt expression in differentiating Th17 cells, thereby causing a prominent reduction in the expression of pro-inflammation cytokines. During the generation of Th17 cells, IL-23/IL-23R signaling can induce RORγt, which further increases IL-23R expression and IL-23 subsequently synergize with TGF-β to conduct the transcription of IL-17 and other Th17 lineage cytokines [38, 39]. With regard to the IFN-γ^+^ IL-17^+^ cells, T cells lacking IL-23R failed to develop into these cells [40]. After tIL12rβ1/Fc treatment, the expression of IL23R mRNA was also decreased along with the decline of STAT3 phosphorylation and RORγt expression, further confirming the rescuing role of tIL12rβ1/Fc during Th17 cell development.

NF-κB is responsible for various biological processes, including the stimulation of inducible nitric oxide synthase (iNOS) [41, 42]and the regulation of pro-inflammatory cytokine IL-6 [43]. Given the importance of NF-κB in the production of inflammatory cytokines and the involvement in Th17 cell development, we examined the effects of tIL12rβ1/Fc on NF-κB signaling and provided strong evidence that tIL12rβ1/Fc can block NF-κB signaling via regulation of IκBα expression and subsequently reduced NF-κB–regulated gene products including IL-6, TNF-α and iNOS. The hematopoietic growth factor, GM-CSF, is also important in the development of EAE and MS [44–46]. Given that the production of GM-CSF is induced by IL-23 and dependent on the activity of the IL-12-IL-23 receptor complex and RORγt [45], the reduction of RORγt through blocking the interaction of IL-23 by tIL12rβ1/Fc and the decline of GM-CSF expression in CNS and spleens in this study strongly illustrate the feasibility and therapeutic potential of using tIL12rβ1/Fc for effective treatment of MS and other autoimmune diseases.

Compared to monoclonal antibodies, especially ustekinumab, the present study indicated that tIL12rβ1/Fc actually exhibits advantageous features in the EAE model. Unlike the ineffectiveness by ustekinumab in rodents EAE, tIL12rβ1/Fc is efficacious in the amelioration of mice EAE model. A significant attenuation of demyelination in stimulated brains and spinal cords of tIL12rβ1/Fc-treated mice was found. However, markedly different degree of demyelination could not be observed in the brains between the antibody-treated and vehicle groups [47]. In addition, the patterns of intracellular IL-12-p40 expression within the CNS white matter did not show difference between antibody- and vehicle-treated animals [48]. In contrast, we observed a remarkable suppression of p40 concentration in lesion CNS as well as in peripheral lymphoid organs after tIL12rβ1/Fc treatment, suggesting that targeting p40 by tIL12rβ1/Fc not only directly blocks the function of released IL-12/IL-23, but also reduces the actual production of IL-12-p40. These effects by tIL12rβ1/Fc could be highly beneficial for disease therapy, and strongly imply the potential on the effectiveness for the treatment of MS and other human autoimmune diseases.

Between EAE and MS, there are several discrepancies to prevent straightforward translation of the efficacy on EAE model to clinical settings. Although the factors for these translational failures are multiple and complicated, a very important fact is that the animal models such as EAE cannot exactly reflect the complex diseases in humans, especially the autoimmune-related ones such as MS [2, 49]. Given that more disease-oriented animal models and sophisticated technologies are needed to develop, alternative animal models currently available for autoimmune diseases, for example, collagen-induced arthritis (CIA) or systemic lupus erythematosus (SLE) should be evaluated to assess the effectiveness of the novel molecule of tIL12rβ1/Fc. These future investigations will not only gain more insights into the molecular mechanism of tIL12rβ1/Fc, but will also expand the scope and potential utility of this molecule.

In conclusion, we developed a novel fusion protein tIL12rβ1/Fc and found that tIL12rβ1/Fc bound the p40 subunit of IL-12/23. tIL12rβ1/Fc effectively ameliorated MOG_35–55_-induced EAE via reducing Th1- and Th17-polarized pro-inflammatory cytokines and suppressing inflammation and demyelination in the focused parts. tIL12rβ1/Fc reduced RORγt and T-bet expression. These results suggest that tIL12rβ1/Fc could be a novel fusion protein to ameliorate MS and other autoimmune diseases.

## MATERIALS AND METHODS

### Construction, expression and purification of tIL12rβ1/Fc

Based on the amino acid sequence of human IL12rβ1 receptor (Genbank NP_005526) and human IgG1 Fc (Genbank AEV43323.1), the human truncated IL12rβ1 receptor (tIL12rβ1) gene containing a signal peptide sequence (708 bp) and the human IgG1 Fc fragment (699 bp) was obtained by RT-PCR from human spleen cDNA library (Biomics, China) using the primers F1, F2 and F3, F4 (Table 2), respectively. After purification with Tian-quick midi purification kit (Tiangen, China), the tIL12rβ1 was ligated with Fc gene using overlap PCR extension with the primers F1 and F4. The constructed tIL12rβ1/Fc fragment was digested with *Xho* I and *Hind* III (Takara, China) and then inserted into the eukaryotic expression vector pcDNA3.1(+) (Invitrogen, USA). *E. coli* DH5α (Novagen, Germany) was transformed with this recombinant plasmid and the following selection was conducted on Luria-Bertani (LB) broth supplemented with 0.1 mg/mL ampicillin (Sigma, USA).

**Table 2:** The PCR primers for preparation of fused tIL12rβ1/Fc gene

Name	Sequence
F1	5′ 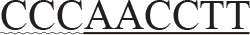 ATGGAGCCGCTGGTGA 3′(*Hind* III)
F2	5′ GGGGTTTTCAGGGGGAAC 3′
F3	5′ GTTCCCCCTGAAAACCCCACAAAGGGCCC TTCTGTG 3′
F4	5′ 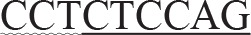 TCACTTGCCGGGGGAC 3′ (*Xho* I)

“
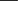
” indicates the enzyme site and “
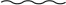
” denotes the corresponding protective base.

After verification of correct sequences by restriction digestion and DNA sequencing (Invitrogen, USA), the plasmids for cell transfection were prepared using EndoFree Maxi Plasmid Kit (Tiangen, China) and then linearized with *Pvu* I (Takara, China). Next, the linearized plasmids were transfected into the CHO-K1 cell line (ATCC, USA) at the logarithmic growth phase using electric transfection. After 24 h cell culture, 400 μg/mL G418 sulfate (Invitrogen, USA) was added into the medium for the selection of monoclonal cell lines. Stably transfected CHO cell lines were identified using ELISA and RT-PCR methods.

Later, the medium was changed to the SFM medium (Gibco, USA) without FBS and protein production was carried out for an additional 96 h after. Cell culture was harvested by centrifugation at 12,000 rpm for 20 min. The resultant supernatant was filtered through a 0.45-μm filter and loaded onto a Protein A Sepharose column (GE Healthcare, USA) pre-equilibrated with binding buffer (20 mmol/L NaH_2_PO4, 20 mmol/L Na_2_HPO4, pH 7.0) at a flow rate of 1 mL/min. Then, the unbound fraction was removed and the resin was washed with 10 column volumes of the binding buffer. Proteins specifically bound to the resin were eluted with 0.1 mol/L Glycine, pH 2.8, and then immediately neutralized with 1 mol/L Tris HCl pH 9.0. The purified protein was identified by SDS-PAGE and Western Blotting.

### Assay of binding specificity

The specificity of tIL12rβ1/Fc protein to human IL-12/IL-23 was examined using a direct ELISA-based receptor binding assay as previously described [50]. The OD_450nm_ absorbance was plotted against tIL12rβ1/Fc concentrations and EC50 values were fitted with a single-site binding model using Prism 5 software (GraphPad, USA).

### Animals used in the experiments

Eight- to twelve-week-old female C57BL/6 mice (Comparative Medicine Centre of Yangzhou) were used in all animal experiments. Mice were kept in a conventional, pathogen-free facility at the Medical School of Southeast University. All animal procedures were in accordance with the Guidelines for the Care and Use of Laboratory Animals as adopted and promulgated by the United States National Institutes of Health, and were approved by the Jiangsu Provincial Experimental Animal Manage Committee under the Contract SCXK 2012(su)-0004.

### Induction and treatment of EAE

Myelin oligodendrocyte glycoprotein (MOG) peptide (MEVGWYRSPFSRVVHLYRNGK, MOG_35–55_) synthesized by GL Biochem, China was dissolved in PBS at 2 mg/mL and emulsified in an equal volume of CFA consisting of IFA (Sigma-Aldrich, USA) plus 4 mg/mL heat-inactivated *Mycobacterium tuberculosis* (strain H37 RA, Difco). 200 μl emulsions were injected s.c. into each mouse over two different sites in the flank. EAE induction was performed using 200 ng Pertussis toxin in 200 μl PBS for each mouse *via* i.v. (tail vein), immediately and 2 d after MOG_35–55_ immunization. Adjuvant control group was set up with equivalent PBS instead of MOG_35–55_ as a negative control. For the treatment of EAE, tIL12rβ1/ Fc or vehicle (saline) was administered *via* tail vein injection at 2.5 mg/kg, and Cyclosporin A (CsA) (Sigma-Aldrich, USA) was administered i.g. at 1.5 mg/kg daily as a positive control. The regimen starts 3 d before (preventive) or 9 d after (treatment) the immunization. Mice were examined every day and clinical assessment of EAE was scored using the standard scale ranging from 0 to 5 as follows: 0, no clinical signs; 0.5, partially limp tail; 1, paralyzed tail; 2, loss in coordinated movement; hind limb paresis; 2.5, one hind limb paralyzed; 3, both hind limbs paralyzed; 3.5, Hind limbs paralyzed; weakness in forelimbs; 4, forelimbs paralyzed; and 5, moribund or death.

### Histopathological analysis

Spinal cords and brains for histological analysis were dissected from mice and immediately fixed in 4% paraformaldehyde. Paraffin-embedded 5–10 um sections of the tissues were stained with Hematoxylin and Eosin (H&E) or immunohistochemistry for CD4 and then examined by light microscopy. Briefly, inflammation was scored as follows: 0, none; 1, a few inflammatory cells; 2, organization of perivascular infiltrates; and 3, increasing severity of perivascular cuffing with extension into the adjacent tissue. Demyelination was scored as follows: 0, none; 1, rare foci; 2, a few areas of demyelination; and 3, large (confluent) areas of demyelination [51].

### Purification of cells

Mice were perfused with 30 mL PBS via the heart before the isolation of spinal cords and brains. The tissues were filtered through 70 μm cell strainers (BD, USA) to yield single-cell suspensions in IMDM containing 10% FBS (invitrogen, USA). CNS mononuclear cells (MNCs) were purified using a Percoll (GE Healthcare, USA) gradient (70/37%).

Spleens and lymph nodes extracted from C57BL/6 mice were teased through sterilized 70 μm cell strainers (BD, USA) to obtain single-cell suspensions in IMDM containing 10% FBS (invitrogen, USA) medium. Red blood cells were lysed with RBC lysis buffer (eBioscience, USA). CD4^+^ T cells were purified by using anti-CD4 magnetic beads (Miltenyi biotech, Germany).

### Differentiation of Th cells

For mouse Th1 and Th17 differentiation, naive CD4^+^ T cells were stimulated with plate-bound anti-mCD3 (5 μg/mL, eBioscience, USA) and soluble anti-mCD28 (1 μg/mL, eBioscience, USA) under Th17-polarizing conditions (10 ng/mL mIL-6, R&D, USA, 1 ng/mL mTGF-β, R&D, USA and 10 ng/mL mIL-23, R&D, USA) or Th1-polarizing conditions (10 ng/mL mIL-12, R&D, USA and 10 mg/mL anti–mIL-4, BD, USA) in the presence or absence of tIL12rβ1/Fc (10 μg/mL and 20 μg/mL). For intracellular cytokine detection, cells were re-stimulated with 1 μl/mL GolgiStop (BD, USA), 20 ng/mL PMA (Sigma, USA) and 1 μg/mL ionomycin (Sigma, USA) for another 4–6 h after 72- hour initial activation.

To induce human Th1 and Th17 development, venous blood was collected from a healthy donator by a trained nurse under aseptic conditions into sodium heparincoated Vacutainer tubes (BD, USA) in Zhongda hospital. Human peripheral blood mononuclear cells (PBMCs) were isolated from leukocyte-rich buffy coats by density gradient centrifugation using Histopaque (Sigma, USA) and then polarized with plate-bound anti-hCD3 (5 μg/mL, eBioscience, USA) and soluble anti-hCD28 (1 μg/mL, eBioscience, USA) under Th17-polarizing conditions (10 ng/mL hIL-β, 10 ng/mL hIL-6, R&D, USA, 1 ng/mL hTGF-β, R&D, USA and 10 ng/mL hIL-23, R&D, USA) or Th1-polarizing conditions (10 ng/mL hIL-12, R&D, USA and 10 mg/mL anti–hIL-4, BD, USA) in the presence or absence of tIL12rβ1/Fc (10 μg/mL and 20 μg/mL). For intracellular cytokine detection, cells were re-stimulated with 1 μl/mL GolgiStop (BD, USA), 20 ng/mL PMA (Sigma, USA) and 1 μg/mL ionomycin (Sigma, USA) for another 4–6 h after 72- hour initial activation.

### Measurement of cytokines

Cytokines were measured using commercially available kits as follows: mIL-12/IL-23 p40 (Dakewe, China), mIL-17A (Dakewe, China), mIL-22 (Biolegend, USA), mIFN-γ (Dakewe, China), mGM-CSF (Cusabio, China), hIL-17A (Dakewe, China) and hIFN-γ (Dakewe, China).

### Flow cytometry

To analyze MOG-specific Th1 and Th17 cells, splenocytes, lymph nodes cells or CNS-infiltrating MNCs were stimulated with 20 μg/ml MOG peptide for 24 h, followed by stimulation with 20 ng/mL PMA (Sigma, USA) and 1 μg/mL ionomycin (Sigma, USA) in the presence of 1 μL/mL GolgiStop (BD, USA) for the last 4–6 h before staining. Cells were first collected, washed, and incubated with Fc block (BD, USA) on ice for 20 min. After washing with staining buffer (BD, USA), the cells were incubated with mAbs against the cell-surface markers for 30 min at 4°C in dark. After washing twice with staining buffer, cells were fixed and permeabilized using Cytofix/Cytoperm solution (BD, USA) for 30 min at 4°C in dark. Then, cells were stained for intracellular cytokines with corresponding mAb diluted in Perm wash solution for 30 min at room temperature in dark. Cells was incubated with anti-CD4 and then intracellular cytokines were detected with anti-IFN-γ and anti-IL17A for Th cells. For the analysis of Th1, Th17 and Th1/Th17 cells using flow cytometry, the IFN-γ single-positive (IL-17^−^) cells were defined as Th1 cells whereas the IL-17 single-positive (IFN-γ^−^) cells were defined as Th17 cells and the IL-17^+^ IFN-γ^+^ double producers were defined as Th1/Th17 cells. The detection of regulatory T (Treg) cells was carried out using the Regulatory T Cell Staining kit (BD, USA), according to the manufacturer's instructions. FACS analysis was performed on a Becton-Dickinson FACSCalibur (BD, USA) and data were analyzed using Flowjo software (Tree Star Int, USA). All staining procedures were performed with following fluorochrome-conjugated Abs: Percp-Cy5.5 anti-mCD4 (BD, USA), APC anti-mCD4 (BD, USA), PE anti-mIL17a (BD, USA), FITC anti-mIFN-γ (BD, USA), PE-anti-mFoxp3 (BD, USA), PE-anti-human IFN-γ (eBioscience, USA), APC-anti-human CD4 (BD, USA), PE-anti-human IL17A (BD, USA). PE rat IgG1 (BD, USA), FITC rat IgG1 (BD, USA) and PE rat IgG2a (BD, USA) were used as isotype controls.

### Quantitative real-time PCR

Gene expression of Th1 and Th17 differentiation factors were determined by quantitative real-time PCR using pre-designed primers by the comparative method of relative quantitation (ΔΔCt). Mouse β-actin gene was used as an internal control for sample normalization. Total RNA was isolated from the cell pellets using RNAprep pure cell kit (Tiangen, China), and the first-strand cDNA was synthesized using TransScript First-Strand cDNA Synthesis SuperMix (Transgen Biotech, China). The quantitative RT-PCR was performed on an ABI Step one Plus Instrument (Applied biosystem, USA) using SYBRgreen Master Mix (Applied biosystem, USA) under standard thermocycler conditions. Sequences of PCR primer pairs are summarized in Table 3.

**Table 3:** Sequences of Q-PCR primer pairs

Name	Sequence (5′-3′)
mIL-17a forward	CAGGGAGAGCTTCATCTGTGT
mIL-17a reverse	GCTGAGCTTTGAGGGATGAT
mRORγt forward	ACCTCTTTTCACGGGAGGA
mRORγt reverse	TCCCACATCTCCCACATTG
mIFN-γ forward	ATCTGGAGGAACTGGCAAAA
mIFN-γ reverse	TTCAAGACTTCAAAGAGTCTGAGGTA
mT-bet forward	CAAGTGGGTGCAGTGTGG
mT-bet reverse	GGTGGACATATAAGCGGTTCC
mβ-actin forward	TAAGGCCAACCGTGAAAAG
mβ-actin reverse	ACCAGAGGCATACAGGGACA
hRORγt forward	TGAGAAGGACAGGGAGCCAA
hRORγt reverse	CCACAGATTTTGCAAGGGATCA
hT-bet forward	TGTGGTCCAAGTTTAATCAGCA
hT-bet reverse	TGACAGGAATGGGAACATCC
hIL-17 forward	TGGGAAGACCTCATTGGTGT
hIL-17 reverse	GGATTTCGTGGGATTGTGAT
hIFN-γ forward	TCGGTAACTGACTTGAATGTCCA
hIFN-γ reverse	TCGCTTCCCTGTTTTAGCTGC
hβ-actin forward	CCAACCGCGAGAAGATGA
hβ-actin reverse	CCAGAGGCGTACAGGGATAG
mIL-23R forward	AGAGACACTGATTTGTGGGAAA
mIL-23R reverse	GTTCCAGGTGCATGTCATGTT
miNOS forward	CTTTGCCACGGACGAGAC
miNOS reverse	TCATTGTACTCTGAGGGCTGAC
mTNF-α forward	CAGGCGGTGCCTATGTCTC
mTNF-α reverse	CGATCACCCCGAAGTTCAGTAG
mIL-6 forward	GCTACCAAACTGGATATAATCAGGA
mIL-6 reverse	CCAGGTAGCTATGGTACTCCAGAA

### Western Blotting

Proteins were extracted with RIPA Lysis Buffer (Thermo, USA) containing proteinase inhibitors (Thermo, USA) and phosphatase inhibitors (Thermo, USA) and then resolved on 10% SDS-PAGE. Western blotting was performed by transferring the proteins onto polyvinylidene difluoride membranes (Millipore, Germany) using a TransBlot system (Bio-Rad, USA). The membranes were washed in ddH_2_O and then blocked with 5% milk in Tris-buffered saline supplemented with 0.1% Tween 20 (TBST) for 2 h at room temperature. After that, the membranes were incubated overnight at 4°C with antibodies against phosphorylated STAT3 (Cell Signaling, USA), STAT3 (Cell Signaling, USA), phosphorylated STAT4 (Cell Signaling, USA), STAT4 (Cell Signaling, USA), phosphorylated IκBα (Santa Cruz, USA), IκBα (Santa Cruz, USA), phosphorylated p65 (Santa Cruz, USA), p65 (Santa Cruz, USA) or β-actin (Santa Cruz, USA) followed by the incubation with HRP-conjugated secondary antibodies for another 1 h at room temperature. After incubation with the secondary antibodies, signals were detected using West Pico Chemiluminescent Substrate (Pierce, USA) according to the manufacturer's instructions.

### Statistical analysis

Results are expressed as the mean ± SD. All data are the results of at least 3 independent experiments. The unpaired *t* test or one way ANOVA with Dunnett's multiple comparison test was used to test statistical significance and a *P* values of <0.05 was considered statistically significant.

## SUPPLEMENTARY FIGURES



## References

[1] Lu Y, Chen B, Song J-H, Zhen T, Wang B-Y, Li X, Liu P, Yang X, Zhang Q-L, Xi X-D (2013). Eriocalyxin B ameliorates experimental autoimmune encephalomyelitis by suppressing Th1 and Th17 cells. Proceedings of the National Academy of Sciences.

[2] Constantinescu CS, Farooqi N, O'Brien K, Gran B (2011). Experimental autoimmune encephalomyelitis (EAE) as a model for multiple sclerosis (MS). British Journal of Pharmacology.

[3] Costa GL, Sandora MR, Nakajima A, Nguyen EV, Taylor-Edwards C, Slavin AJ, Contag CH, Fathman CG, Benson JM (2001). Adoptive immunotherapy of experimental autoimmune encephalomyelitis via T cell delivery of the IL-12 p40 subunit. The Journal of Immunology.

[4] Moldovan IR, Rudick RA, Cotleur AC, Born SE, Lee J-C, Karafa MT, Pelfrey CM (2003). Interferon gamma responses to myelin peptides in multiple sclerosis correlate with a new clinical measure of disease progression. Journal of neuroimmunology.

[5] Steinman L (2007). A brief history of T(H)17, the first major revision in the T(H)1/T(H)2 hypothesis of T cell-mediated tissue damage. Nature medicine.

[6] Cua DJ, Sherlock J, Chen Y, Murphy CA, Joyce B, Seymour B, Lucian L, To W, Kwan S, Churakova T (2003). Interleukin-23 rather than interleukin-12 is the critical cytokine for autoimmune inflammation of the brain. Nature.

[7] Kroenke MA, Carlson TJ, Andjelkovic AV, Segal BM (2008). IL-12-and IL-23-modulated T cells induce distinct types of EAE based on histology, CNS chemokine profile, and response to cytokine inhibition. The Journal of experimental medicine.

[8] Stromnes IM, Cerretti LM, Liggitt D, Harris RA, Goverman JM (2008). Differential regulation of central nervous system autoimmunity by TH1 and TH17 cells. Nature medicine.

[9] Langrish CL, Chen Y, Blumenschein WM, Mattson J, Basham B, Sedgwick JD, McClanahan T, Kastelein RA, Cua DJ (2005). IL-23 drives a pathogenic T cell population that induces autoimmune inflammation. The Journal of experimental medicine.

[10] Bettelli E, Carrier Y, Gao W, Korn T, Strom TB, Oukka M, Weiner HL, Kuchroo VK (2006). Reciprocal developmental pathways for the generation of pathogenic effector TH17 and regulatory T cells. Nature.

[11] Veldhoen M, Hocking RJ, Atkins CJ, Locksley RM, Stockinger B (2006). TGFβ in the context of an inflammatory cytokine milieu supports de novo differentiation of IL-17-producing T cells. Immunity.

[12] Annunziato F, Cosmi L, Santarlasci V, Maggi L, Liotta F, Mazzinghi B (2007). Phenotypic and functional features of human Th17 cells. The Journal of experimental medicine.

[13] Annunziato F, Romagnani S (2010). The transient nature of the Th17 phenotype. European journal of immunology.

[14] Duhen R, Glatigny S, Arbelaez CA, Blair TC, Oukka M, Bettelli E (2013). Cutting edge: the pathogenicity of IFN-gamma-producing Th17 cells is independent of T-bet. Journal of immunology.

[15] Abromson-Leeman S, Bronson RT, Dorf ME (2009). Encephalitogenic T cells that stably express both T-bet and ROR gamma t consistently produce IFNgamma but have a spectrum of IL-17 profiles. Journal of neuroimmunology.

[16] Yeilding N, Szapary P, Brodmerkel C, Benson J, Plotnick M, Zhou H, Goyal K, Schenkel B, Giles-Komar J, Mascelli MA, Guzzo C (2011). Development of the IL-12/23 antagonist ustekinumab in psoriasis: past, present, and future perspectives. Annals of the New York Academy of Sciences.

[17] McInnes IB, Kavanaugh A, Gottlieb AB, Puig L, Rahman P, Ritchlin C, Brodmerkel C, Li S, Wang Y, Mendelsohn AM (2013). Efficacy and safety of ustekinumab in patients with active psoriatic arthritis: 1 year results of the phase 3, multicentre, double-blind, placebo-controlled PSUMMIT 1 trial. The Lancet.

[18] Hayden MS, Ghosh S (2011). NF-κB in immunobiology. Cell research.

[19] Kodama S, Davis M, Faustman DL (2005). The therapeutic potential of tumor necrosis factor for autoimmune disease: a mechanistically based hypothesis. Cell Mol Life Sci.

[20] Maini RN, Taylor PC, Szechinski J, Pavelka K, Broell J, Balint G (2006). Double-blind randomized controlled clinical trial of the interleukin-6 receptor antagonist, tocilizumab, in European patients with rheumatoid arthritis who had an incomplete response to methotrexate. Arthritis and rheumatism.

[21] Krueger GG, Langley RG, Leonardi C, Yeilding N, Guzzo C, Wang Y, Dooley LT, Lebwohl M (2007). A human interleukin-12/23 monoclonal antibody for the treatment of psoriasis. New England Journal of Medicine.

[22] McInnes IB, Kavanaugh A, Gottlieb AB, Puig L, Rahman P, Ritchlin C, Brodmerkel C, Li S, Wang YH, Mendelsohn AM, Doyle MK, Grp PS (2013). Efficacy and safety of ustekinumab in patients with active psoriatic arthritis: 1 year results of the phase 3, multicentre, double-blind, placebo-controlled PSUMMIT 1 trial. Lancet.

[23] Martin R (2008). Neutralisation of IL12 p40 or IL23 p40 does not block inflammation in multiple sclerosis. Lancet Neurol.

[24] Segal BM, Constantinescu CS, Raychaudhuri A, Kim L, Fidelus-Gort R, Kasper LH (2008). Repeated subcutaneous injections of IL12/23 p40 neutralising antibody, ustekinumab, in patients with relapsing-remitting multiple sclerosis: a phase II, double-blind, placebo-controlled, randomised, dose-ranging study. The Lancet Neurology.

[25] Brahmachari S, Pahan K (2008). Role of cytokine p40 family in multiple sclerosis. Minerva medica.

[26] Pahan K, Sheikh FG, Liu X, Hilger S, McKinney M, Petro TM (2001). Induction of nitric-oxide synthase and activation of NF-κB by interleukin-12 p40 in microglial cells. Journal of Biological Chemistry.

[27] Jana M, Dasgupta S, Saha RN, Liu X, Pahan K (2003). Induction of tumor necrosis factor-α (TNF-α) by interleukin-12 p40 monomer and homodimer in microglia and macrophages. Journal of neurochemistry.

[28] Mondal S, Roy A, Pahan K (2009). Functional blocking monoclonal antibodies against IL-12p40 homodimer inhibit adoptive transfer of experimental allergic encephalomyelitis. The Journal of Immunology.

[29] Wang X, Wilkinson VL, Podlaski FJ, Wu Cy, Stern AS, Presky DH, Magram J (1999). Characterization of mouse interleukin-12 p40 homodimer binding to the interleukin-12 receptor subunits. European journal of immunology.

[30] Kebir H, Ifergan I, Alvarez JI, Bernard M, Poirier J, Arbour N, Duquette P, Prat A (2009). Preferential recruitment of interferon-gamma-expressing TH17 cells in multiple sclerosis. Ann Neurol.

[31] Okamoto S, Fujiwara H, Nishimori H, Matsuoka K, Fujii N, Kondo E, Tanaka T, Yoshimura A, Tanimoto M, Maeda Y (2015). Anti-IL-12/23 p40 antibody attenuates experimental chronic graft-versus-host disease via suppression of IFN-gamma/IL-17-producing cells. Journal of immunology.

[32] Annunziato F, Cosmi L, Liotta F, Maggi E, Romagnani S (2009). Human Th17 cells: are they different from murine Th17 cells?. European journal of immunology.

[33] Kurschus F, Croxford A, Heinen A, Wörtge S, Ielo D, Waisman A (2010). Genetic proof for the transient nature of the Th17 phenotype. Eur J Immunol.

[34] Szabo SJ, Kim ST, Costa GL, Zhang X, Fathman CG, Glimcher LH (2000). A novel transcription factor, T-bet, directs Th1 lineage commitment. Cell.

[35] Rengarajan J, Szabo SJ, Glimcher LH (2000). Transcriptional regulation of Th1/Th2 polarization. Immunology today.

[36] Yang XO, Panopoulos AD, Nurieva R, Chang SH, Wang D, Watowich SS, Dong C (2007). STAT3 regulates cytokine-mediated generation of inflammatory helper T cells. Journal of Biological Chemistry.

[37] Ivanov II, McKenzie BS, Zhou L, Tadokoro CE, Lepelley A, Lafaille JJ, Cua DJ, Littman DR (2006). The orphan nuclear receptor ROR gamma t directs the differentiation program of proinflammatory IL-17(+) T helper cells. Cell.

[38] Ouyang W, Kolls JK, Zheng Y (2008). The biological functions of T helper 17 cell effector cytokines in inflammation. Immunity.

[39] Zhou L, Ivanov II, Spolski R, Min R, Shenderov K, Egawa T, Levy DE, Leonard WJ, Littman DR (2007). IL-6 programs TH-17 cell differentiation by promoting sequential engagement of the IL-21 and IL-23 pathways. Nature immunology.

[40] Ahern P, Schiering C, Buonocore S, McGeachy M, Cua D, Maloy K, Powrie F (2010). Interleukin-23 drives intestinal inflammation through direct activity on T cells. Immunity.

[41] Yamamoto Y, Gaynor RB (2001). Therapeutic potential of inhibition of the NF-κB pathway in the treatment of inflammation and cancer. Journal of Clinical Investigation.

[42] Zhu J, Paul WE (2010). Peripheral CD4+ T-cell differentiation regulated by networks of cytokines and transcription factors. Immunological reviews.

[43] Ma X, Reynolds SL, Baker BJ, Li X, Benveniste EN, Qin H (2010). IL-17 enhancement of the IL-6 signaling cascade in astrocytes. The Journal of Immunology.

[44] El-Behi M, Ciric B, Dai H, Yan Y, Cullimore M, Safavi F, Zhang GX, Dittel BN, Rostami A (2011). The encephalitogenicity of T(H)17 cells is dependent on IL-1- and IL-23-induced production of the cytokine GM-CSF. Nature immunology.

[45] Codarri L, Gyülvészi G, Tosevski V, Hesske L, Fontana A, Magnenat L, Suter T, Becher B (2011). RORγt drives production of the cytokine GM-CSF in helper T cells, which is essential for the effector phase of autoimmune neuroinflammation. Nature immunology.

[46] Noster R, Riedel R, Mashreghi MF, Radbruch H, Harms L, Haftmann C, Chang HD, Radbruch A, Zielinski CE (2014). IL-17 and GM-CSF expression are antagonistically regulated by human T helper cells. Science translational medicine.

[47] Hart BAt, Brok HPM, Remarque E, Benson J, Treacy G, Amor S, Hintzen RQ, Laman JD, Bauer J, Blezer ELA (2005). Suppression of Ongoing Disease in a Nonhuman Primate Model of Multiple Sclerosis by a Human-Anti-Human IL-12p40 Antibody. The Journal of Immunology.

[48] Brok HP, van Meurs M, Blezer E, Schantz A, Peritt D, Treacy G, Laman JD, Bauer J, Bert A (2002). Prevention of experimental autoimmune encephalomyelitis in common marmosets using an anti-IL-12p40 monoclonal antibody. The Journal of Immunology.

[49] Croxford AL, Kurschus FC, Waisman A (2011). Mouse models for multiple sclerosis: historical facts and future implications. Biochimica et Biophysica Acta (BBA)-Molecular Basis of Disease.

[50] Guo W, Luo C, Wang C, Zhu Y, Wang X, Gao X, Yao W (2012). Protection against Th17 Cells Differentiation by an Interleukin-23 Receptor Cytokine-Binding Homology Region. PloS one.

[51] Li H, Nourbakhsh B, Ciric B, Zhang G-X, Rostami A (2010). Neutralization of IL-9 ameliorates experimental autoimmune encephalomyelitis by decreasing the effector T cell population. The Journal of Immunology.

