# Supplementary material for: A novel human truncated IL12rβ1-Fc fusion protein ameliorates experimental autoimmune encephalomyelitis via specific binding of p40 to inhibit Th1 and Th17 cell differentiation
Source: Oncotarget. 2015 Sep 4;6(30):28539–55. doi: 10.18632/oncotarget.5164 (PMC4745676; doi:10.18632/oncotarget.5164)
Supplement: Supplementary file 1 [file oncotarget-06-28539-s001.pdf]

SUPPLEMENTARY FIGURES

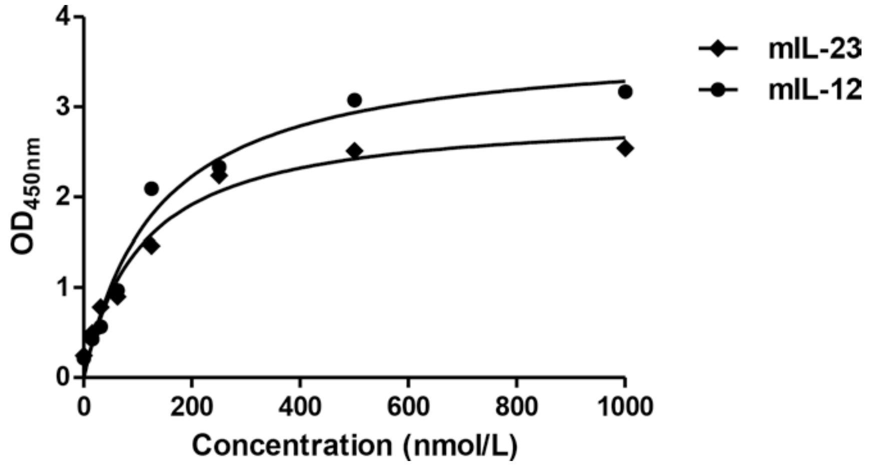

Supplementary Figure S1: Binding affinity of tIL12rβ1/Fc protein to mouse IL-12/IL-23. Binding affinity of tIL12rβ1/Fc protein to mouse IL-12 and mouse IL-23 were examined by direct binding ELISA to give EC_{50} values of 134.4 and 107.1 nmol/L, respectively.

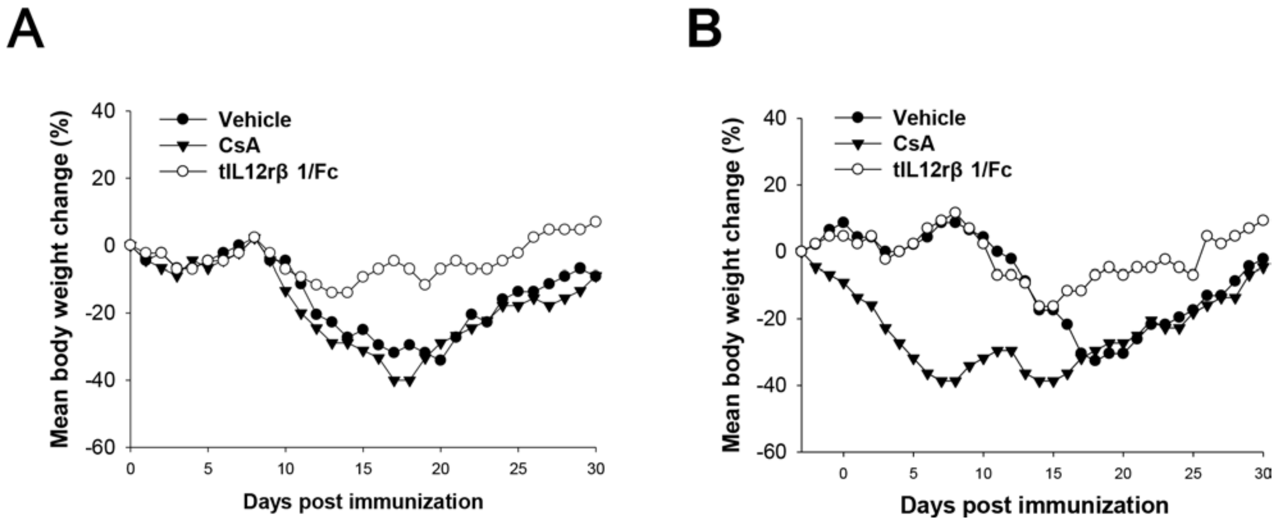

Supplementary Figure S2: tIL12rβ1/Fc does not lead to body weight loss. The efficacy of tIL12rβ1/Fc was also determined by the percent change in body weight under therapeutic **A.** and preventive protocol **B.**
